# Effects of a four week detraining period on physical, metabolic, and inflammatory profiles of elderly women who regularly participate in a program of strength training

**DOI:** 10.1186/s11556-020-00244-8

**Published:** 2020-08-26

**Authors:** Carolina P. Celestrin, Guilherme Z. Rocha, Angelica M. Stein, Dioze Guadagnini, Rafael M. Tadelle, Mario J. A. Saad, Alexandre G. Oliveira

**Affiliations:** 1 Department of Physical Education, Bioscience Institute, Sao Paulo State University (UNESP), Rio Claro, SP 13506-900 Brazil; 2 Department of Internal Medicine, State University of Campinas, Campinas, Brazil; 3 The Human Performance Research Group, Technological Federal University of Paraná, Curitiba, Brazil

**Keywords:** Aging, Inflammation, Physical exercise

## Abstract

**Background:**

Human aging has innumerable health implications, including loss of muscle mass and increased circulating inflammatory markers. Resistance exercise in the elderly can prevent muscle mass loss and improve the inflammatory profile. Conversely, detraining can reverse this picture. Thus, there is a strong need for studies with the elderly population to clarify the real impacts of a training interruption. Therefore, the objective of this study was to analyze the inflammatory profile of resistance trained elderly women after 4 weeks of detraining.

**Methods:**

Seventeen elderly women with regular participation in an exercise program participated in the study. Body mass index (BMI), physical activity level assessments, total cholesterol and its fractions, triglycerides, glycemia and insulin blood levels, IL-1β, IL-4, IL-6, IL-10, IL-13, TNF-α, IFNγ, and MCP-1 were assessed before and after the detraining protocol.

**Results:**

The 4 week detraining period decreased physical fitness without altering body mass and BMI**.** The short detraining period was able to induce some metabolic disturbances in elderly women who regularly participate in a program of strength training, such as increasing HOMA-IR (0.72 ± 0.14 to 0.81 ± 0.23; *p* = 0.029), and increasing total blood cholesterol (178.21 ± 23.64 to 220.90 ± 64.98 mg/dL; *p* = 0.008) and LDL fraction (111.79 ± 21.09 to 155.33 ± 60.95 mg/dL; *p* = 0.048). No alteration in levels of inflammatory cytokines was observed, however, this detraining period significantly reduced IL-13 (44.84 ± 100.85 to 35.84 ± 78.89 pg/mL; *p* = 0.031) a Th2 cytokine that induces M2 macrophage polarization.

**Conclusions:**

These data demonstrate that even a short period of detraining is harmful for elderly women who regularly participate in a program of strength training, since it impairs physical performance, insulin sensitivity and cholesterol metabolism.

## Background

Physical inactivity has been considered an independent risk factor for development of several diseases such as cardiovascular diseases, dyslipidemia, insulin resistance, type 2 diabetes, and some cancers [1–4]. Furthermore, aging has been strongly associated with chronic subclinical inflammation, which in turn increases the risk of several chronic diseases [5]. A negative correlation has been observed between strength and inflammatory markers in the elderly [6]. On the other hand, resistance exercise training can attenuate systemic inflammation as well as promoting muscle mass maintenance during aging [5, 7]. The anti-inflammatory effects of exercise may have an important role since higher levels of pro-inflammatory markers are associated with muscle catabolism [8–10]. Indeed, resistance exercise training can reduce lean mass loss, and therefore promotes maintenance of autonomy in the elderly [11–13].

Elevated levels of pro-inflammatory markers are strongly associated with increased risk of developing diabetes, cardiovascular diseases, and some cancers [5, 14]. Moreover, obesity and visceral adiposity show positive correlation with elevated levels of pro-inflammatory cytokines [15, 16], and adipose tissue inflammation seems to contribute with muscle wasting [17]. Likewise, studies have associated some chronic diseases to elevated pro-inflammatory cytokine levels as a consequence of the aging process [5, 18, 19]. In addition, circulating levels of IL-6 and TNF-α are higher in the elderly than in young or middle-aged individuals [15, 20]. Such an increase of circulating pro-inflammatory cytokines observed during aging may result from a combination of imbalanced immune function, presence of disease condition, reduced levels of physical activity, and increased fat mass [5, 21–23]. Conversely, several groups have found a negative correlation between physical activity levels and pro-inflammatory markers, i.e., higher levels of physical activity are associated with lower levels of circulating inflammatory markers in the elderly [24–29]. In addition, strength trained elderly women presented reduced levels of TNF-α, when compared to non-exercised elderly women [30]. Furthermore, a review of researches analyzing the effects of exercise on inflammation in the elderly suggested that increasing physical activity levels may be an important tool to lower inflammation in aging [31]. Thus, it seems clear that long-term exercise training can be a useful therapy to reduce inflammation during the aging process.

In parallel, elderly people are more likely to interrupt their physical activity than younger individuals due to disease, injuries, and incidence of depression [32]. Studies have shown that training interruption resulted in significant loss of upper and lower limb muscle strength in postmenopausal women [33] and reduced levels of aerobic and strength performance [34]. In addition to these changes related to strength performance, detraining may also affect metabolic biomarkers and endothelial health [35]. Since increased metabolic disturbances are highly prevalent in older people, mainly in those with obesity, it becomes important to understand the impacts of detraining in the elderly.

Despite the evidence showing the benefits of exercise training for the elderly, there is limited research regarding the detrimental effects that detraining may exert in the inflammatory and metabolic profile of this group. Thus, the current study aimed to investigate whether a 4-week detraining period was able to blunt the benefits of 4 months strength training in elderly women.

## Methods

The study was approved by the Institutional Ethics Review Board at the Sao Paulo State University (UNESP) and was carried out in accordance with the Declaration of Helsinki from 1964 and revised in 2000. All participants included in the study signed a written informed consent and provided a medical authorization for physical exercise practice.

### Participants

Seventeen physically active elderly women participated in the study. Participants were included in the study if they met the following inclusion criteria: a minimum of 60 years old and regular participation of 3 times a week in the resistance training provided by the State University of São Paulo (UNESP - Rio Claro). All volunteers accomplished at least 75% of the exercise sessions for 4 months (March to June). The training session was structured with stretching exercises in warm up, and resistance training as the major part. Each session lasted 1 h, with resistance training of 40 min. All participants performed the same exercises on a rotating system, being repeated on the 3 days of the week (Monday, Wednesday and Friday). The resistance training consists of three sets of 15 repetition maximum in the following exercises: lat pulldowns (latissimus dorsi), triceps pushdown (triceps), pec deck fly (chest), leg press (quadriceps), dumbbell curls (biceps), lateral raises (shoulders), standing calf raises (gastrocnemius). With respect to training load, participants were encouraged to choose a load that allowed them to perform up to 15 repetitions. Thus, when the participants were able to perform more than 15 repetitions, the load was adjusted and under supervision of a physical educator. Participants with any mental, neurological, muscular, and/or osteo-articular contraindications that limited or made impossible the accomplishment of the protocol of exercises during the classes and evaluation, or who missed 1 evaluation, or who had more than 2 absences in the month were excluded from the study. No participants were diabetic, nor had other metabolic diseases, or were in use of medications that interfered with insulin activity or glucose and fatty acid metabolism.

### Detraining

On the day after the last exercise session, blood samples and physical assessment for base line data were collected. Blood was sampled from elderly women in the morning (7:00 to 8:00 am) after a 10-h overnight fasting. The physical assessment was carried out in the afternoon. Following this, all participants were oriented to not perform any physical exercise during the next 4 weeks. After this period the participants returned to the Lab for collection of blood samples and physical parameters evaluation in the same conditions applied for baseline to assess the detraining effects in these variables (Figure S1). We can assure that all volunteers neither attended other physical activity programs nor performed physical exercise during this detraining period since we questioned them on this matter. Thus, the detraining of the current study consists of 4 weeks without performing any physical exercise.

### Anthropometric variables

Body mass was obtained using a mechanical scale with an accuracy of 0.1 kg (Welmy, SP, Brazil), and height was determined with a stadiometer (Sanny, SP, Brazil), fixed to the wall, with an accuracy of 0.01 m as previously described [36].

### Metabolic variables

Total cholesterol and its fractions, triglycerides, glycemia, and insulin blood levels were assessed before and after the detraining protocol. Blood was sampled from elderly women in the morning after an overnight fasting in 2 moments, approximately 24 h after the last training session and 1 day before starting over again, i.e., following 4 weeks of detraining. As the training session had mild to moderate intensity, we decided to collect the samples 1 day after the last training session to avoid significant alterations disturbances in metabolic and inflammatory parameters, as previously performed [37, 38]. The blood was collected in tubes containing EDTA and centrifuged at 1100 g for 20 min at 4 °C. Serum samples were stored at − 20 °C until analyzed. Insulin and adiponectin levels were assessed using a commercial ELISA kit according to the manufacturer’s protocol, while triglycerides, total cholesterol and its fraction were assessed by the colorimetric method. Glycemia was evaluated using a glucometer (Optium Xceeed – Abbot, Berkshire, England).

### HOMA-IR

HOMA-IR was used as a proxy measure of whole body insulin sensitivity using the following formula (fasting serum insulin (μU/ml) × fasting plasma glucose (mmol l^− 1^)/22.5) [39].

### Multiplex bead Array platform

Blood samples were collected and centrifuged, and the blood serum samples were stored at − 20 °C until use. IL-1β, IL-4, IL-6, IL-10, IL-13, TNF-α, IFNγ, and MCP-1 were quantified with a Milliplex Map Human Cyotkine/Chemokine Magnetic Bead Panel (Merck Millipore, Germany) according to the manufacturer’s instructions and read on a Luminex Magpix instrument (Luminex, Austin, TX, USA). Data were analyzed with xPONENT 4.2 software (Luminex) as previously described [40].

### Physical activity variables

#### Physical activity level

Physical activity levels were self-reported through the score obtained with the Baecke questionnaire modified for the elderly. The Baecke questionnaire consists of 10 questions related to basic activity, leisure time utilization, and physical activity, as previously described [41].

#### Strength performance

To evaluate the upper limb strength, the test of the American Alliance for Health, Physical Education, Recreation and Dance (AAHPERD) was used. In this test, the participant sits in an armless chair and performs the maximal number of elbow flexion for 30 s. The participants performed the test twice and the higher value of repetition between the two tries is considered to measure the resistance strength [42, 43]. The 30 s chair test was used to assess lower limb performance. The volunteer must sit on a chair with a straight back without arm rests and stand as many times as possible in 30 s [44, 45].

#### Flexibility

The Wells bench test was used as a way to measure muscular flexibility in lower limbs. This test consists of a small wooden apparatus with a metric scale on its surface. The individual sits on the floor with both legs fully extended and with the sole of the foot in one of the grooves, and then extends their hands far as possible in order to measure the score [46].

### Statistical analyses

Participants’ characterizing data (age, mass, height, and anthropometric data) were described as mean and standard deviation. To verify data distribution the Shapiro-Wilk test was used. In addition, outlier cases were calculated from the Grubbs test and then extreme values were excluded from analyzes. In normal distribution situation, the inflammatory profile, functional capacity components, and metabolic analysis of the participants were compared by the T Test (Student’s T-Test) at two moments, pre detraining and post detraining. For non-normal distribution, the Friedman test was used when comparing the pre and post detraining in the aforementioned variables. In the case of a significant difference indicated by the Friedman test, the Wilcoxon test was adopted as equivalent to post hoc. The Cohen’s d was used to verify the magnitude of detraining. A significance level of 5% was accepted for all analyzes.

## Results

### Effects of detraining on physical activity variables

**Fig. 1:**
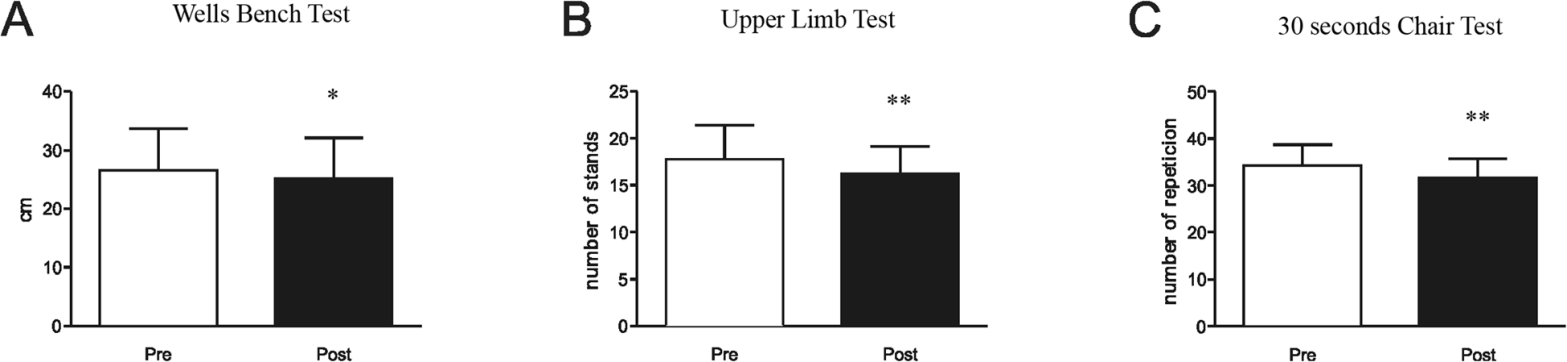
Effects of a detraining period on physical activity levels. **a** Wells bench test, **b** 30 s chair stand test, and **c** upper limb resistance test in two moments before (Pre) and after (Post) a 4 week detraining period. Data are presented as means ± standard deviation, * *P* < 0.05 vs Pre, and **P < 0.01 vs Pre

Table 1 shows age, average program participation, height, mass and body mass index (BMI). No significant change between the baseline (Pre) and Post detraining period (Post) for both body mass and BMI were observed (Table 1). With regard to physical parameters, a significant reduction was observed in strength performance and flexibility after the detraining period as measured by the Wells bench test (26 ± 7 to 25 ± 7 cm; *p* = 0.015, and small effect with *d* = 0.19), 30 s chair (18 ± 4 to 16 ± 3 number of stands; *p* = 0.009, and small effect *d* = 0.46) and upper limb test (34 ± 5 to 32 ± 4 repetitions; *p* = 0.006, and medium effect *d* = 0.57) (Fig. 1a-c, and Supplemental Table 1).

**Table 1:**
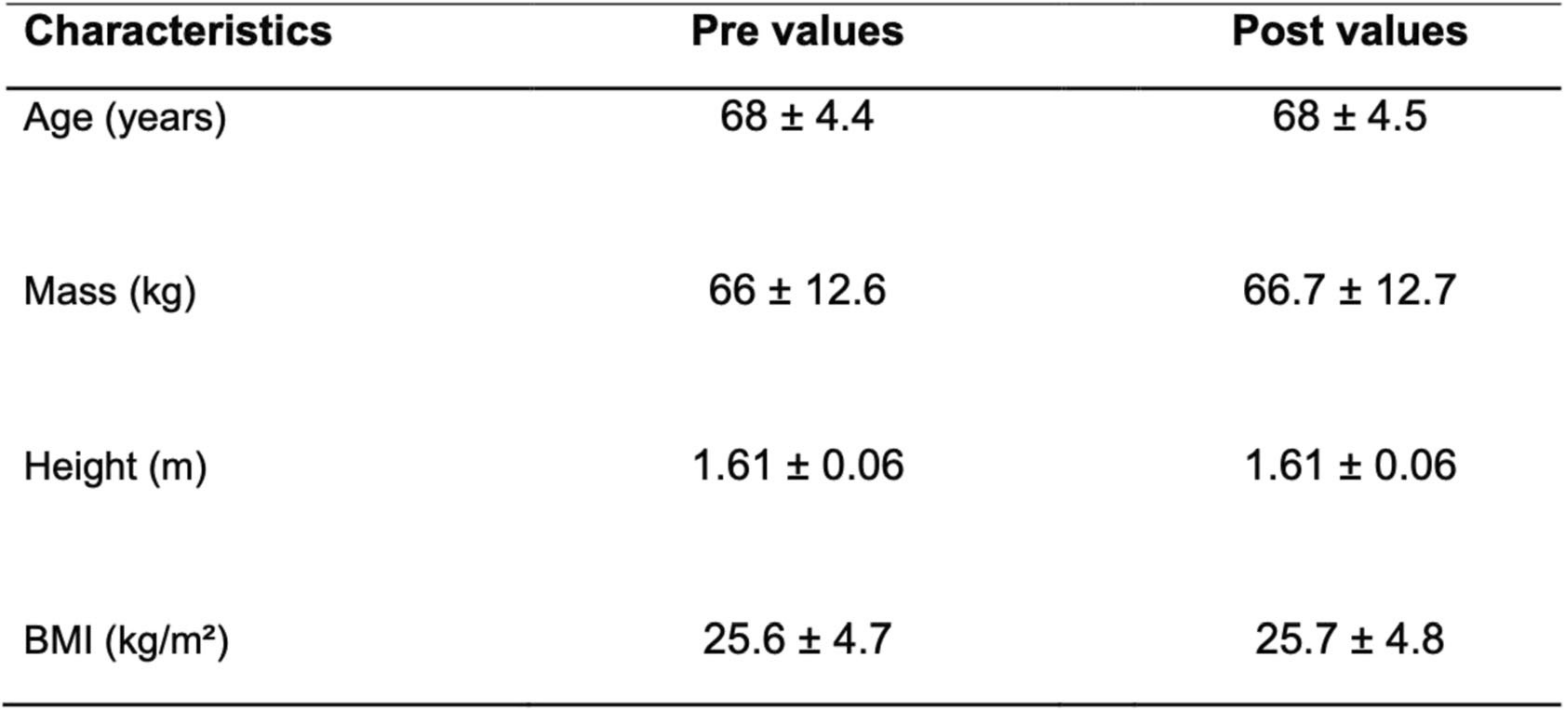
Physical characteristics and age, mean and standard deviation (*n*=17)

*BMI* body mass index

### Metabolic parameters after the 4 week detraining period

**Fig. 2:**
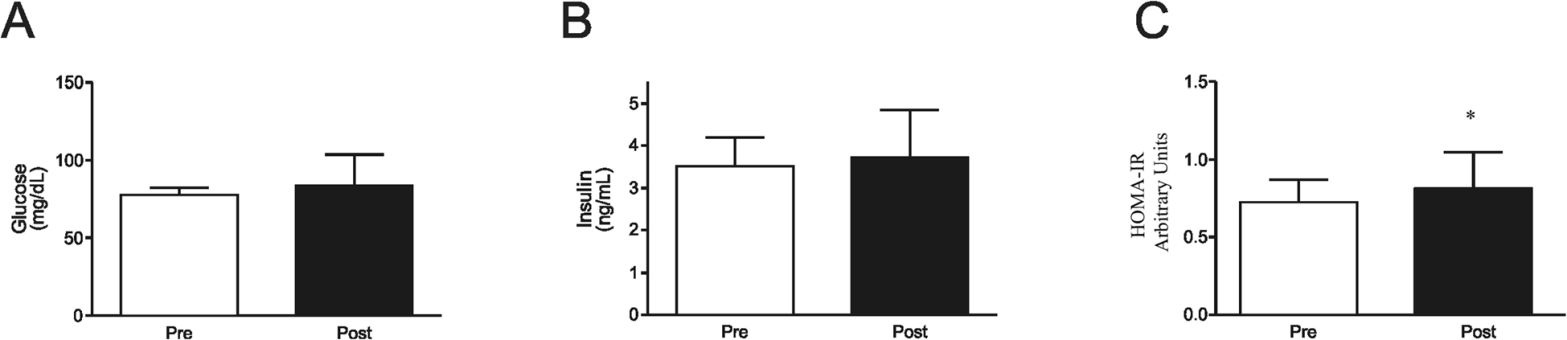
Detraining effects on glucose metabolism. **a** fasting blood glucose, **b** fasting serum insulin, and **c** HOMA-IR in two moments before (Pre) and after (Post) a 4 week detraining period. Data are presented as means ± standard deviation, * *P* < 0.05 vs Pre

**Fig. 3:**
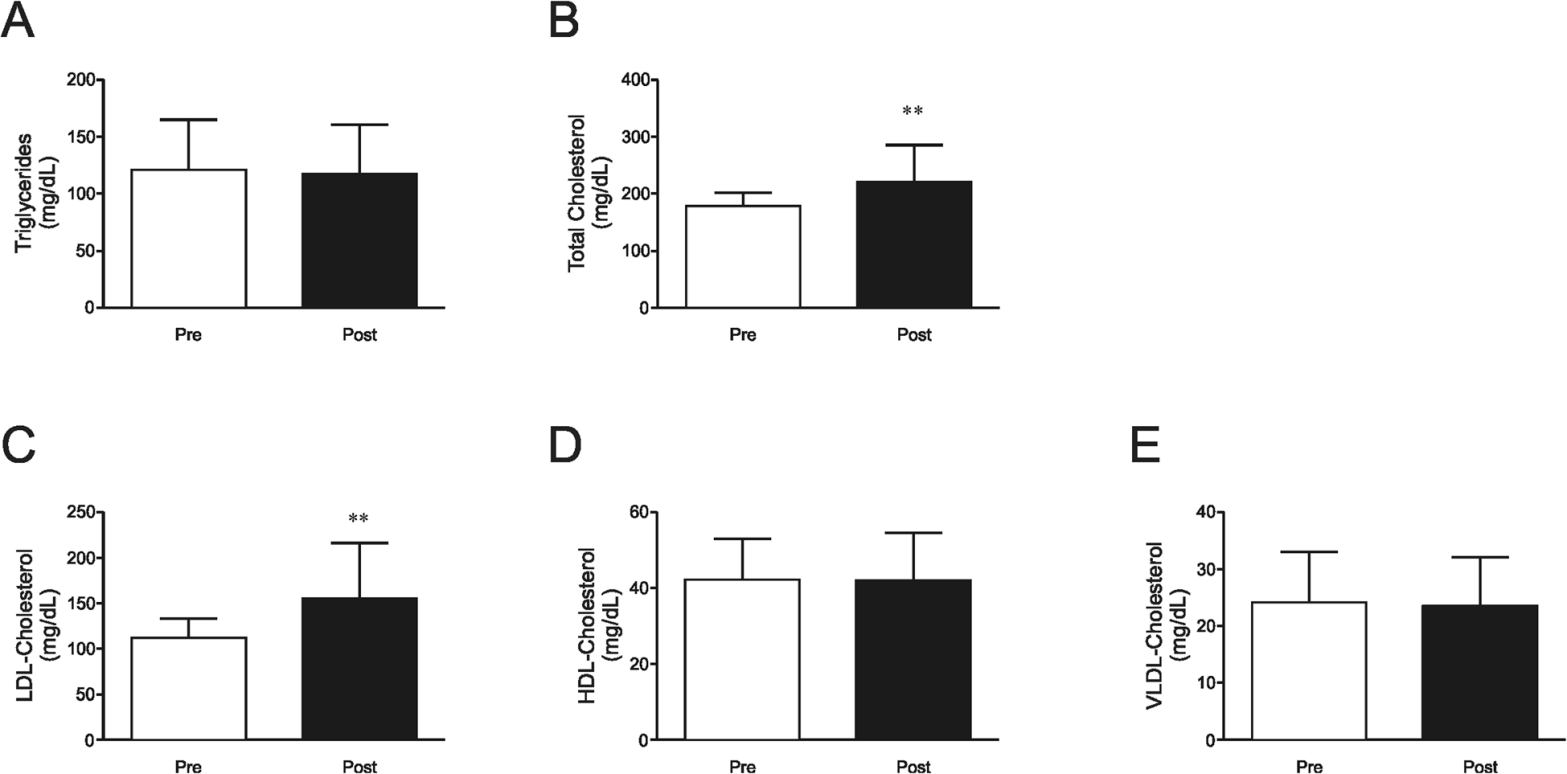
Effects of detraining on lipid profile. **a** fasting blood triglycerides, **b** total cholesterol, **c** LDL cholesterol, **d** HDL cholesterol, and **e** VLDL cholesterol in two moments before (Pre) and after (Post) a 4 week detraining period. Data are presented as means ± standard deviation, ** *P* < 0.01 vs Pre

No significant difference was found following the detraining period for both blood glucose (*p* = 0.26), and insulin (*p* = 0.37) (Fig. 2a and b, and Supplemental Table 1). However, when HOMA-IR, a proxy measure of whole body insulin sensitivity, before and after the detraining period was compared, a significant increase in this index was observed (0.72 ± 0.14 to 0.81 ± 0.23; *p* = 0.029, and small effect with *d* = 0.47) (Fig. 2c, and Supplemental Table 1). We then evaluated the effects of the 4 week detraining period over the lipid profile. With respect to triglyceride levels, no significant change was observed when comparing Pre-detraining and Post-detraining (*p* = 0.46) (Fig. 3a, and Supplemental Table 1). On the other hand, the detraining period resulted in a significant increase in total blood cholesterol (178.21 ± 23.64 to 220.90 ± 64.98 mg/dL; *p* = 0.008, and large effect with *d =* 0.87) and in LDL fraction (111.79 ± 21.09 to 155.33 ± 60.95 mg/dL; *p* = 0.048, and large effect with *d* = 0.95) (Fig. 3b and c, and Supplemental Table 1). However, no significant alteration in HDL (*p* = 0.95) and VLDL (*p* = 0.46) cholesterol fractions were found when comparing the two moments (Fig. 3d and e, and Supplemental Table 1).

### Effects of detraining on the inflammatory status

**Fig. 4:**
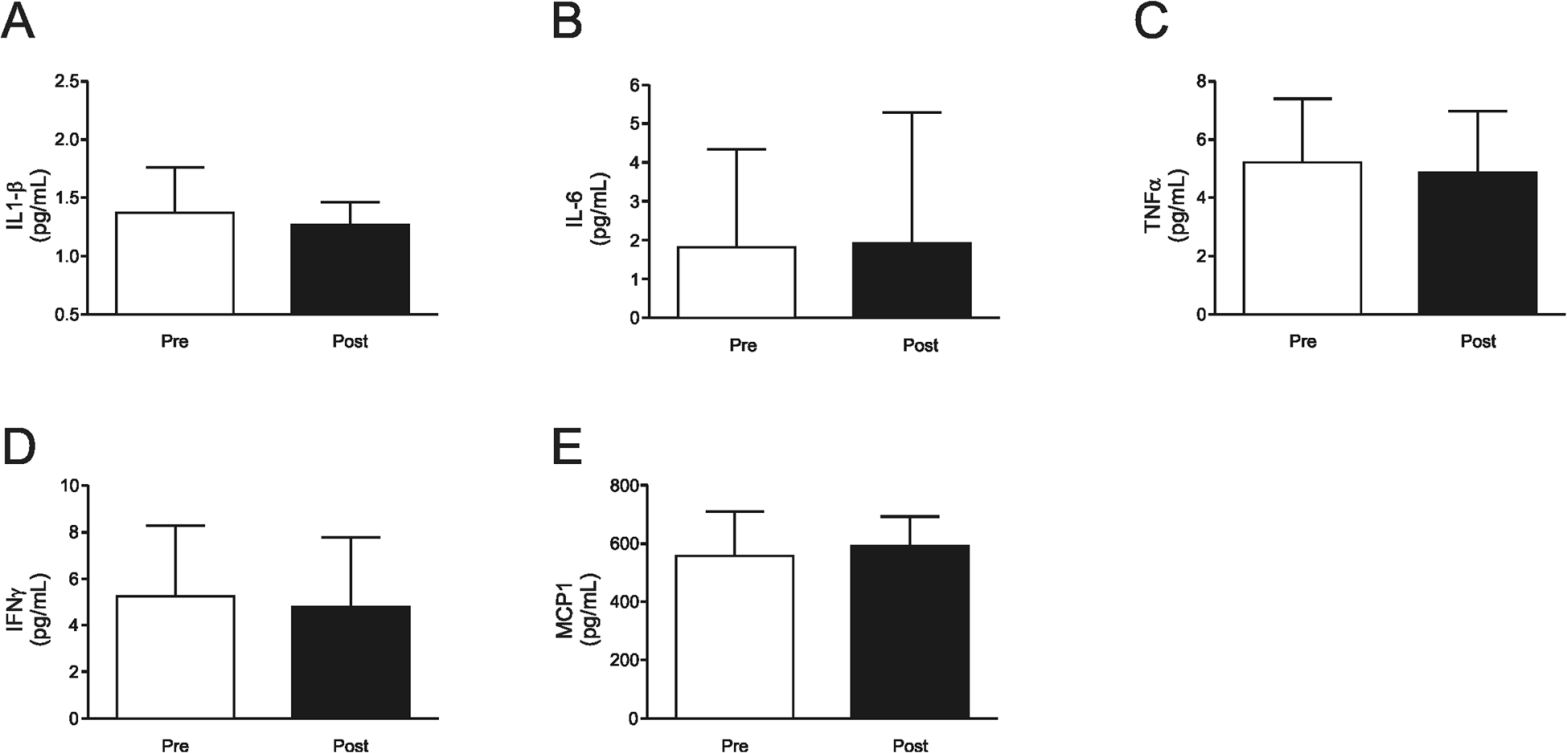
Pro-inflammatory profile after detraining. **a** IL-1β, **b** IL-6, **c** TNF-α, **d** IFN-γ, and **e** MCP-1 in two moments before (Pre) and after (Post) a 4 week detraining period. Data are presented as means ± standard deviation

**Fig. 5:**
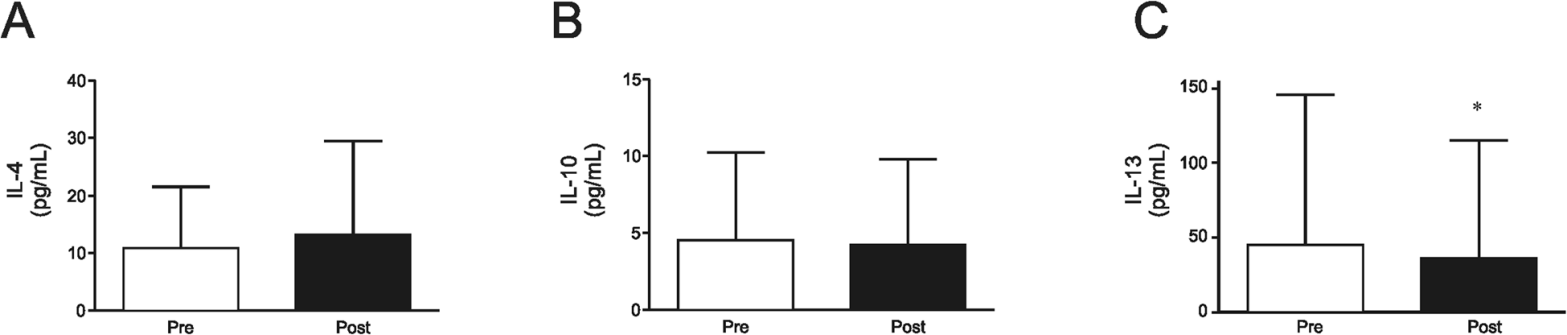
Effects of detraining on anti-inflammatory cytokines. **a** IL-4, **b** IL-10, and **c** IL-13 in two moments before (Pre) and after (Post) a 4 week detraining period. Data are presented as means ± standard deviation, * *P* < 0.05 vs Pre

We subsequently decided to investigate whether this detraining period was able to change the inflammatory profile of elderly women who regularly participate in a program of strength training. For this purpose, a wide investigation in circulating cytokine levels by using a multiplex assay was performed. Regarding inflammatory cytokines, no significant changes were observed for IL-1β (*p* = 0.31), IL-6 (*p* = 0.22), TNF-α (*p* = 0.34), IFNγ (*p* = 0.34), and MCP-1 (*p* = 0.36) after the detraining period (Fig. 4a-e, and Supplemental Table 1). In the same manner, no significant alterations in anti-inflammatory cytokines such as IL-4 (*p* = 0.97) and IL-10 (*p* = 0.27) were observed (Fig. 5a and b, and Supplemental Table 1). However, when another important Th2 cytokine such as IL-13 was assessed, a significant reduction was observed after the detraining period (44.84 ± 100.85 to 35.84 ± 78.89 pg/mL; *p* = 0.03, and small effect with *d* < 0.2) (Fig. 5c, and Supplemental Table 1).

### Effects of a short detraining period in lipopolysaccharide circulating levels in physically active aging women

**Fig. 6:**
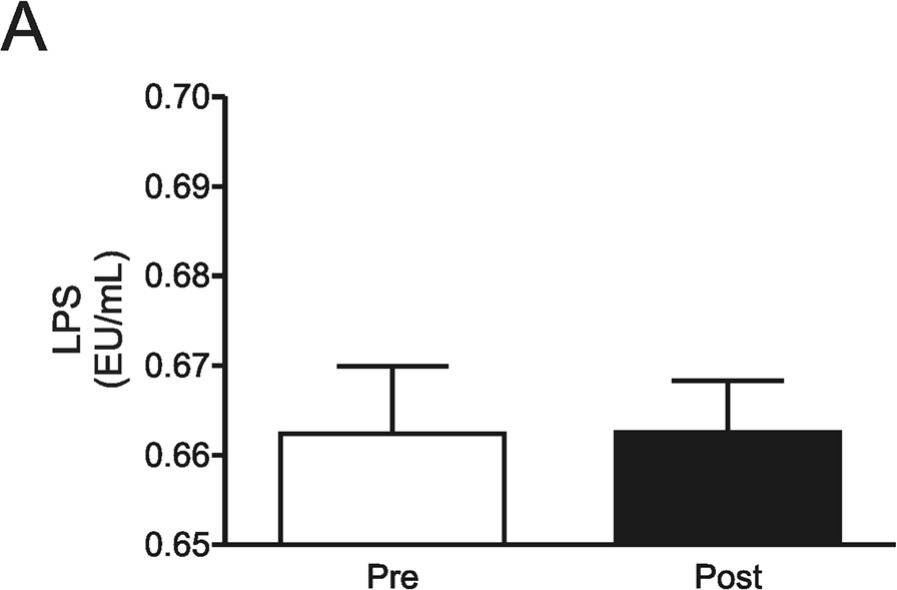
LPS levels after a 4 week detraining period. **a** LPS blood levels in two moments before (Pre) and after (Post) a 4 week detraining period. Data are presented as means ± standard deviation

Since physical inactivity has been associated with increased pro-inflammatory cytokines, we hypothesized that a detraining period may induce an increase in the blood levels of lipopolysaccharide (LPS), which in turn would increase activation of inflammatory pathways in insulin target tissues. However, our data demonstrated that a 4 week period of detraining is not enough to increase circulating LPS levels (*p* = 0.25) (Fig. 6a, and Supplemental Table 1).

## Discussion

In the current study, we demonstrated that a relatively short detraining period of only 4 weeks, was able to impair insulin sensitivity and cholesterol metabolism. Herein, we also observed that this detraining period significantly reduced IL-13, a Th2 cytokine that induces M2 macrophage polarization. However, this detraining period was not able to increase blood levels of pro-inflammatory cytokines and did not result in significant body mass gain or changes in BMI of elderly women who regularly participate in a program of strength training.

In accordance, middle-aged women subjected to Pilates training did not show difference in body mass between the Pre and Post 4 week detraining periods [47]. On the other hand, the research of Gastebois and contributors observed that the same detraining period induces a significant change in body composition as evidenced by reduced lean mass and increased fat mass, despite any change in body weight [48]. Since body composition was not assessed in the current study, we cannot rule out that our elderly women did not present such alterations. In addition, although we strongly recommended that their diets should be unaltered during the 4-week detraining period, it is tempting to speculate that they may have reduced caloric intake, which in turn would contribute to the absence of weight gain in the current study. Thus, it seems evident that assessing body composition and food intake certainly would be of major importance to evaluate the detraining effects on anthropometric parameters in future studies.

Since in the present study elderly women who regularly participate in a program of strength training were evaluated, it would be expected that the 4 week detraining resulted in a significant decrease in physical performance. In this regard, our participants showed reduced performance in flexibility (Wells bench test) as well as in strength performance of lower limbs (30 s chair sit to stand) and upper limbs (upper limb test) when comparing the Post detraining period with baseline. In accordance, a recent study showed that a 12 week detraining period decreases the performance in the Wells bench test, 30 s chair sit to stand and in upper muscle strength performance [49]. Together these findings confirm that detraining, even for a short period such as 4 weeks, induces significant loss in flexibility and strength performance.

The present study demonstrates that a 4-week detraining period may deteriorate the health of elderly women who regularly participate in a program of strength training, as we observed a significant increase in total cholesterol levels, driven by the elevation of its LDL fraction. Also, resistance training is particularly positive for elderly women since this kind of exercise improves strength and prevents muscle mass loss, which, in turn, would be useful to prevent health deterioration [7]. Taking all these into account, it becomes evident that elderly women who regularly participate in a program of strength training should avoid stopping their physical activity regimen for periods longer than 2 to 3 weeks.

Our results showed that the 4-week detraining period did not induced an increase in both glucose and insulin blood levels. Corroborating these results, two studies of independent groups that used the same protocol as ours, i.e. a 4-week detraining period, did not observe any significant difference in both fasting glycemia and insulinemia after detraining [48, 50]. However, when these two parameters are analyzed together, i.e. by using the HOMA-IR index, a proxy measure of whole body insulin sensitivity, we verified a significant reduction in insulin sensitivity of elderly women subjected to a 4 week detraining period. Fatouros and coworkers observed that a 24-week detraining period increased glycaemia and HOMA-IR in trained elderly [51]. Taking the aforementioned into account, we can hypothesize that the detraining influence on metabolic parameters seems to be closely related to its length, i.e., the longer the duration of detraining, the greater the impact on metabolic parameters. Thus, there is still a need for further studies to evaluate the impact of detraining duration on metabolic changes.

The 4-week detraining period did not induce any significant change in pro-inflammatory markers such as TNF-α, IL-1β, IL-6, and MCP-1. These results are consistent with previous studies which also found no increase in TNF-α after a detraining period of 4 weeks [52, 53]. On the other hand, 8 weeks of detraining was able to increase the ratio neutrophil/lymphocyte, an index of systemic inflammation, which also showed a positive correlation with insulin resistance [54]. Taken together these data indicate that the increase of inflammation during a detraining period may occur slowly or even have a late onset, since systemic significant changes can be observed only after 8 weeks. In addition, our results do not allow ruling out the occurrence of changes in the production of pro-inflammatory cytokines at tissue level. Further invasive studies are needed to elucidate the spatial and temporal kinetics of increased inflammation in the course of the detraining process.

Despite not observing an increase in the pro-inflammatory markers, it was observed that the 4-week detraining period resulted in a significant reduction in the circulating levels of IL-13. Such change may have major relevance since this cytokine strongly induces M2 macrophage polarization, that is important to the maintenance of physiological insulin sensitivity [55, 56]. Furthermore, studies have shown that improvements in insulin sensitivity occur due to action of IL-4 and IL-13 in the JAK1/STAT6 pathway and that IL-13 promotes suppression of hepatic glucose production [57, 58]. In this regard it is tempting to speculate that a circulating IL-13 decrease may be interfering with anti-inflammatory responses, and therefore have a role in increasing insulin resistance in the elderly women subjected to a detraining period as observed in the current study. Thus, further studies are necessary to elucidate this point.

The current study presents some limitations. The main limitation is certainly the absence of a control group, i.e., a group of elderly women who continue exercising. The best conduct would be to assess pre and post exercise parameters in both experimental and control groups. However, we did find significant decreases in some physical parameters (flexibility and strength performance) when comparing pre to post exercise, therefore, we believe that the alterations found in this study are most likely a result of the detraining period. Thus, our results may be considered meaningful in-spite of the lack of a control group. Another limitation is the absence of rigorous monitoring of physical activity behavior during the detraining period, to avoid possible interferences of any increase of daily physical activity during the detraining in pro-inflammatory cytokines, or in physical and metabolic parameters. However, we found significant changes in some measurements after the detraining period, which in turn indicated that our group of elderly women avoided performing physical activity during the detraining. The other important limitation is certainly the lack of any nutritional assessment of the participants before, during, or after the detraining period in order to exclude the possible interferences of usual dietary intake, or even the last meal in parameters such as body mass, circulating cytokine levels, LPS levels, and also in HOMA-IR. In addition, the sample size is certainly a limitation that have weakened the statistical power, thus interfering in the size effect and detection of significant differences. Further studies should take into account these factors.

Thus, despite these above limitations, the current study has contributed to its field, since we observed that even a short period of detraining was able to induce metabolic disturbances, which in turn highlighted the importance of avoiding training interruption for elderly women who regularly participate in a program of strength training.

## Conclusion

In summary, we demonstrate that a 4-week detraining period impairs insulin sensitivity and cholesterol metabolism, as evidenced by increased HOMA-IR, and cholesterol LDL fraction, along with a reduction in circulating levels of IL-13, a Th2 response inductor. However, this short period was not able to induce alterations in pro-inflammatory cytokine circulating levels in elderly women who regularly participate in a program of strength training.

## Supplementary information

**Additional file 1.**

## Data Availability

Not applicable.
