# Supplementary material for: Effects of a four week detraining period on physical, metabolic, and inflammatory profiles of elderly women who regularly participate in a program of strength training
Source: Eur Rev Aging Phys Act. 2020 Aug 26;17:12. doi: 10.1186/s11556-020-00244-8 (PMC7450596; doi:10.1186/s11556-020-00244-8)
Supplement: Supplementary file 1 — Additional file 1. [file 11556_2020_244_MOESM1_ESM.pdf]

SUPPLEMENTARY MATERIAL

Figure S1

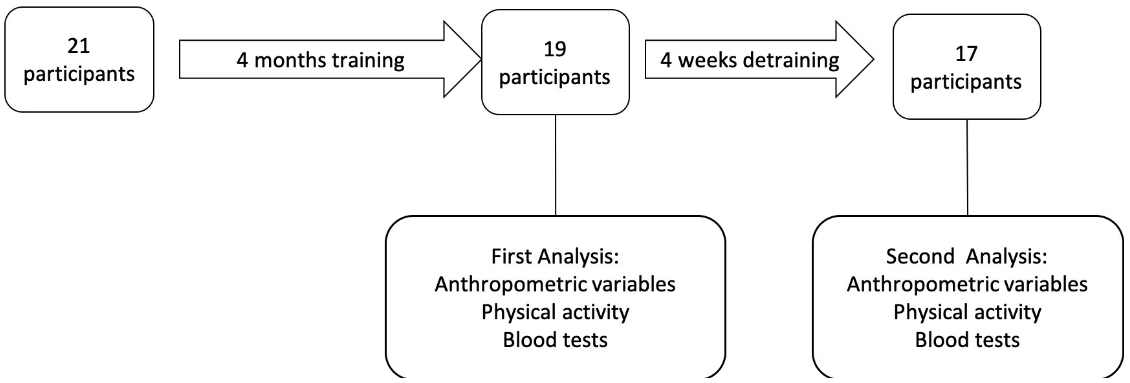

TABLE S1

Variables (unit of measurement)	Pre-training (mean ± SD)	CV%	Post-training (mean ± SD)	CV%	p-value
**Wells Bench test (cm)**	26 ± 7	27.32	25 ± 7	27.97	0.015
**30 s chair stand test (stands)**	18 ± 4	20.44	16 ± 3	17.76	0.009
**Upper limb strength test (repetitions)**	34 ± 5	13.26	32 ± 4	12.65	0.006
**Blood glucose (mg/dL)**	77.65 ± 4.87	6.27	83.67 ± 20.11	24.04	0.26
**Insulin (ng/mL)**	3.50 ± 0.68	19.48	3.71 ± 1.14	30.57	0.37
**HOMA-IR (arbitrary)**	0.72 ± 0.14	19.72	0.81 ± 0.23	29.10	0.029
**Triglycerides (mg/dL)**	120.87 ± 44.17	36.54	117.41 ± 43.26	36.84	0.046
**Total Cholesterol (mg/dL)**	178.21 ± 23.64	13.27	220.90 ± 64.98	29.42	0.008
**LDL Cholesterol (mg/dL)**	111.79 ± 21.09	19.60	155.33 ± 60.95	39.24	0.048
**HDL Cholesterol (mg/dL)**	42.24 ± 10.68	25.28	42.09 ± 12.41	29.49	0.95
**VLDL Cholesterol (mg/dL)**	24.17 ± 8.83	36.54	23.48 ± 8.65	36.84	0.46
**IL1β (pg/mL)**	1.37 ± 0.39	28.57	1.26 ± 0.20	15.80	0.31
**IL6 (pg/mL)**	1.82 ± 2.52	138.42	1.91 ± 3.38	176.86	0.22
**TNFα (pg/mL)**	5.22 ± 2.19	41.97	4.86 ± 2.12	43.70	0.34
**INFγ (pg/mL)**	5.24 ± 3.04	58.06	4.79 ± 2.99	62.46	0.34
**MCP1 (pg/mL)**	558.41 ± 151.16	27.07	592.06 ± 100.09	16.91	0.36
**IL4 (pg/mL)**	10.89 ± 10.70	98.28	13.21 ± 16.31	123.49	0.97
**IL10 (pg/mL)**	4.53 ± 5.71	126.04	4.23 ± 5.55	131.33	0.27
**IL13 (pg/mL)**	44.84 ± 100.85	224.90	35.84 ± 78.89	220.11	0.03
**LPS (EU/mL)**	0.66 ± 0.01	1.14	0.66 ± 0.01	0.87	0.25

LEGEND OF SUPPLEMENTARY FIGURES

Figure S1. Flowchart diagram of the research.
